# Supplementary figures and images for: Brca2, Pds5 and Wapl differentially control cohesin chromosome association and function
Source: PLoS Genet. 2018 Feb 15;14(2):e1007225. doi: 10.1371/journal.pgen.1007225 (PMC5831647; doi:10.1371/journal.pgen.1007225)

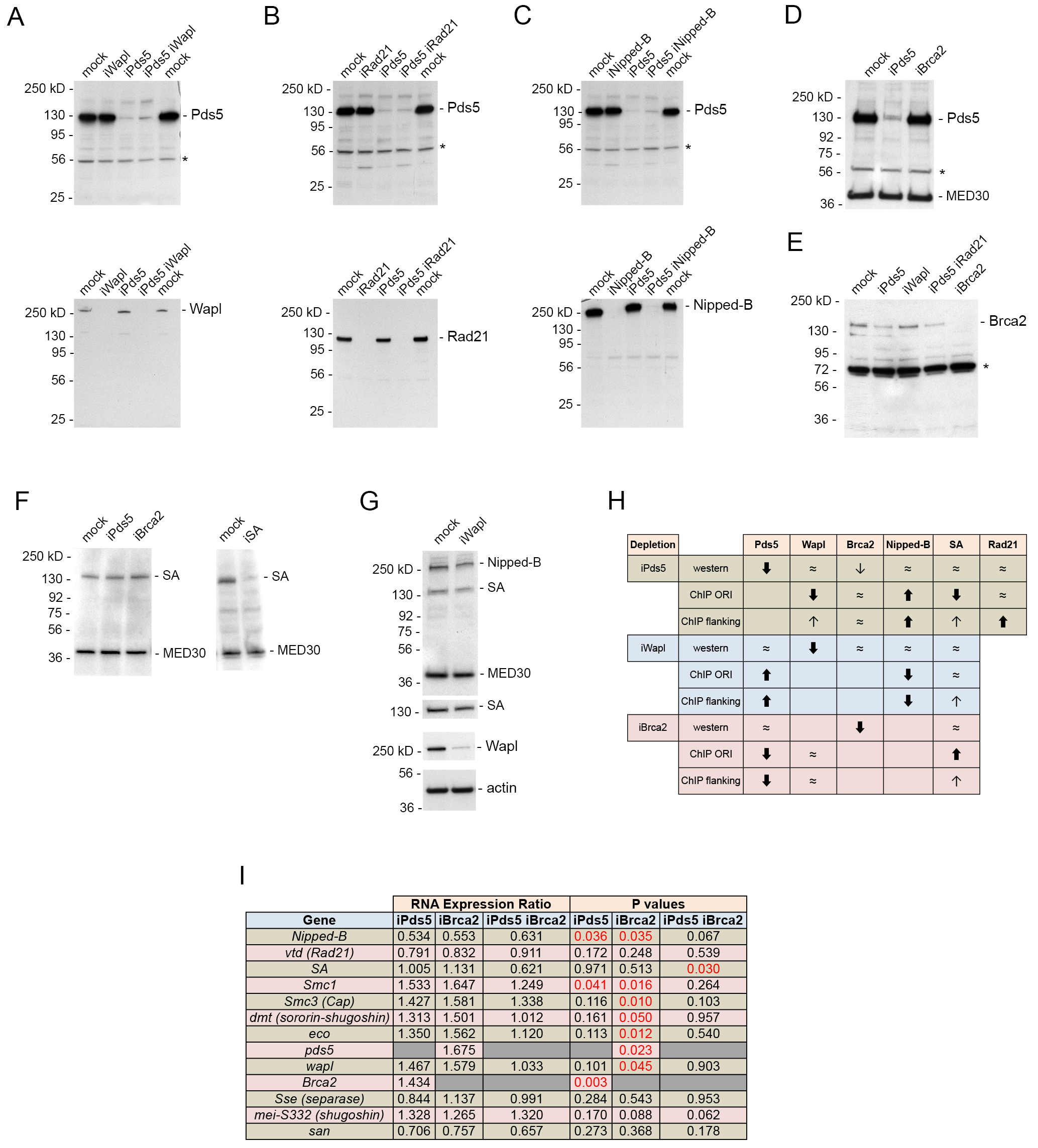

Supplement: S1 Fig — (A) The top and bottom panels are matched western blots of whole cell extracts of cells after the indicated single and double RNAi treatments for 4 to 5 days. The top panel is probed with anti-Pds5 antibody and the bottom panel is probed with anti-Wapl. The asterisk (*) indicates a non-specific band recognized by the Pds5 antibody used as a loading control. (B) Matched western blots with extracts from cells with the indicated single and double RNAi treatments. The top panel was probed with anti-Pds5 and the bottom panel was probed with anti-Rad21. (C) Matched western blots with extracts from cells with the indicated RNAi treatments. The top was probed with anti-Pds5 and the bottom was probed with anti-Nipped-B. (D) Western of extracts of cells after the indicated RNAi treatments (mock, iPds5, iBrca2) probed with anti-Pds5 and anti-MED30 as a loading control. (E) Western of extracts of cells after the indicated RNAi treatments (mock, iPds5, iWapl, iPds5 iRad21, iBrca2) treated probed with anti-Brca2. The asterisk (*) indicates a non-specific band recognized by anti-Brca2. (F) The left western shows extracts of cells with the indicated RNAi treatments (mock, iPds5, iBrca2) probed with anti-SA, and anti-MED30 Mediator subunit. The right panel is a western of extracts from mock-treated cells and cells depleted for SA (iSA) and anti-MED30 to demonstrate SA antibody specificity. (G) The top western shows extracts from mock-treated cells and cells depleted for Wapl (iWapl) probed with anti-Nipped-B, anti-SA, and anti-MED30. The second panel down shows a longer exposure for SA from the same blot. The third panel down shows the same blot when re-probed with anti-Wapl, and the bottom panel when re-probed with anti-actin. (H) Summary of the effects of Pds5, Wapl and Brca2 depletions (iPds5, iWapl, iBrca2) on the levels of the indicated proteins by western blot of whole cell extracts, and ChIP-seq enrichment at replication origin centers (ChIP ORI) or in regions flanking replic [file pgen.1007225.s001.tif]

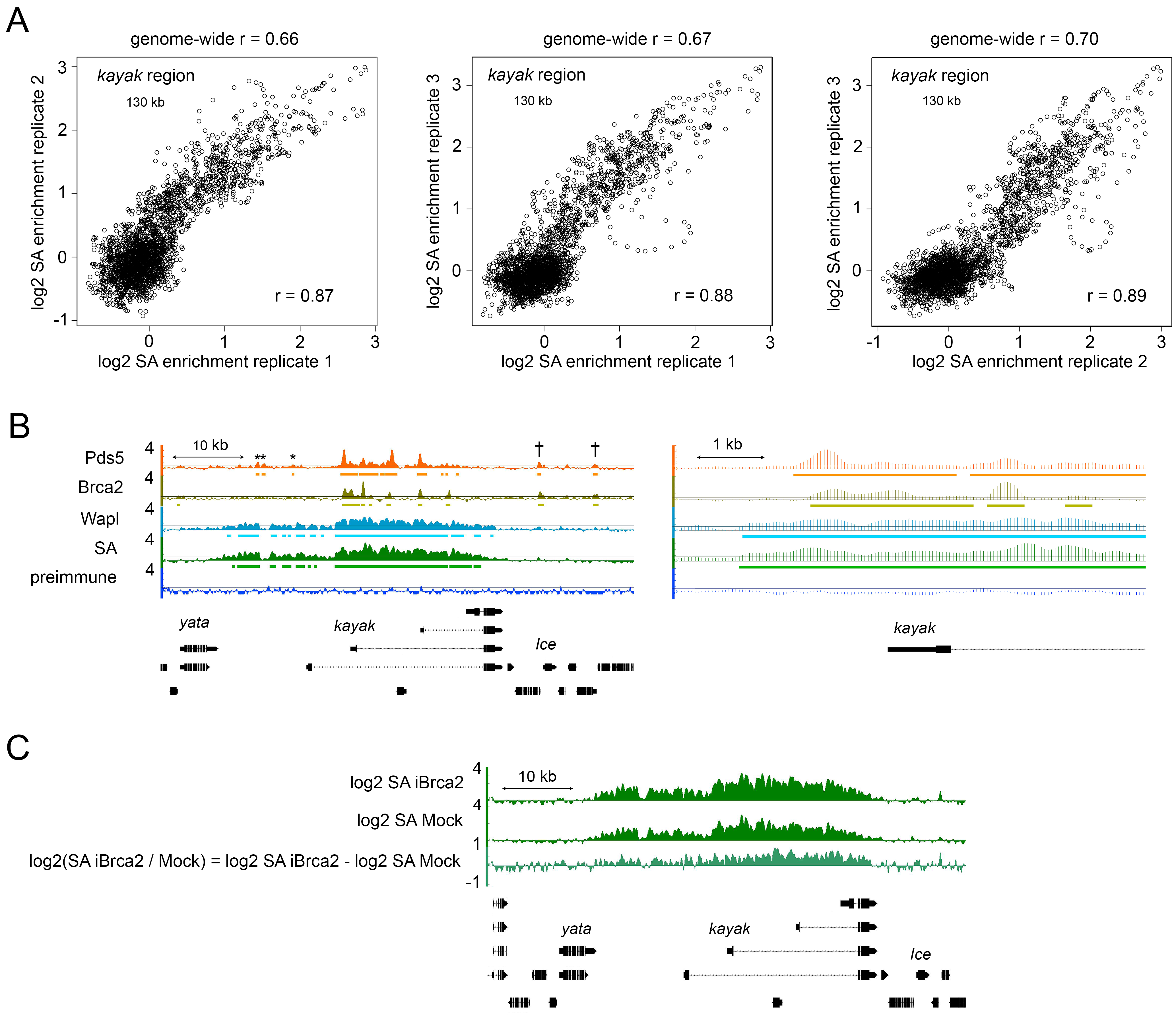

Supplement: S2 Fig — (A) SA ChIP-seq enrichment normalized to input chromatin (>45X genome coverage) every 50 bp across a 130 kilobase kayak region from three independent biological replicate experiments sequenced to at least 10X genome coverage are plotted against each other as examples of the reproducibility of the ChIP-seq method used for these studies. The genome-wide Pearson correlations between the two replicates plotted in each panel are above the plot, and the correlations in the 130 kilobase region surrounding kayak are given in the plot. (B) Genome browser views of Pds5, Brca2, Wapl, SA and preimmune serum ChIP-seq enrichment (log2 values) are shown as an example of the lack of significant enrichment with preimmune serum, indicating a lack of methodological artifacts. Bars underneath the ChIP-seq enrichment plots indicate where enrichment is in the 95 percentile or higher for at least 300 base pairs. Asterisks (*) indicate Pds5 binding sites without significant Brca2 occupancy. Daggers (†) indicate Pds5 –Brca2 binding sites in regions with little cohesin or Wapl. The right panel shows a higher resolution view of one of the active kayak gene promoters, illustrating the ChIP-seq enrichment values every 50 base pairs, simplifying downstream data analysis. (C) Example of an increase in SA enrichment at the kayak locus upon Brca2 depletion (iBrca2). The method used to calculate the fold-change in enrichment every 50 base pairs is the bottom track. (TIF) [file pgen.1007225.s002.tif]

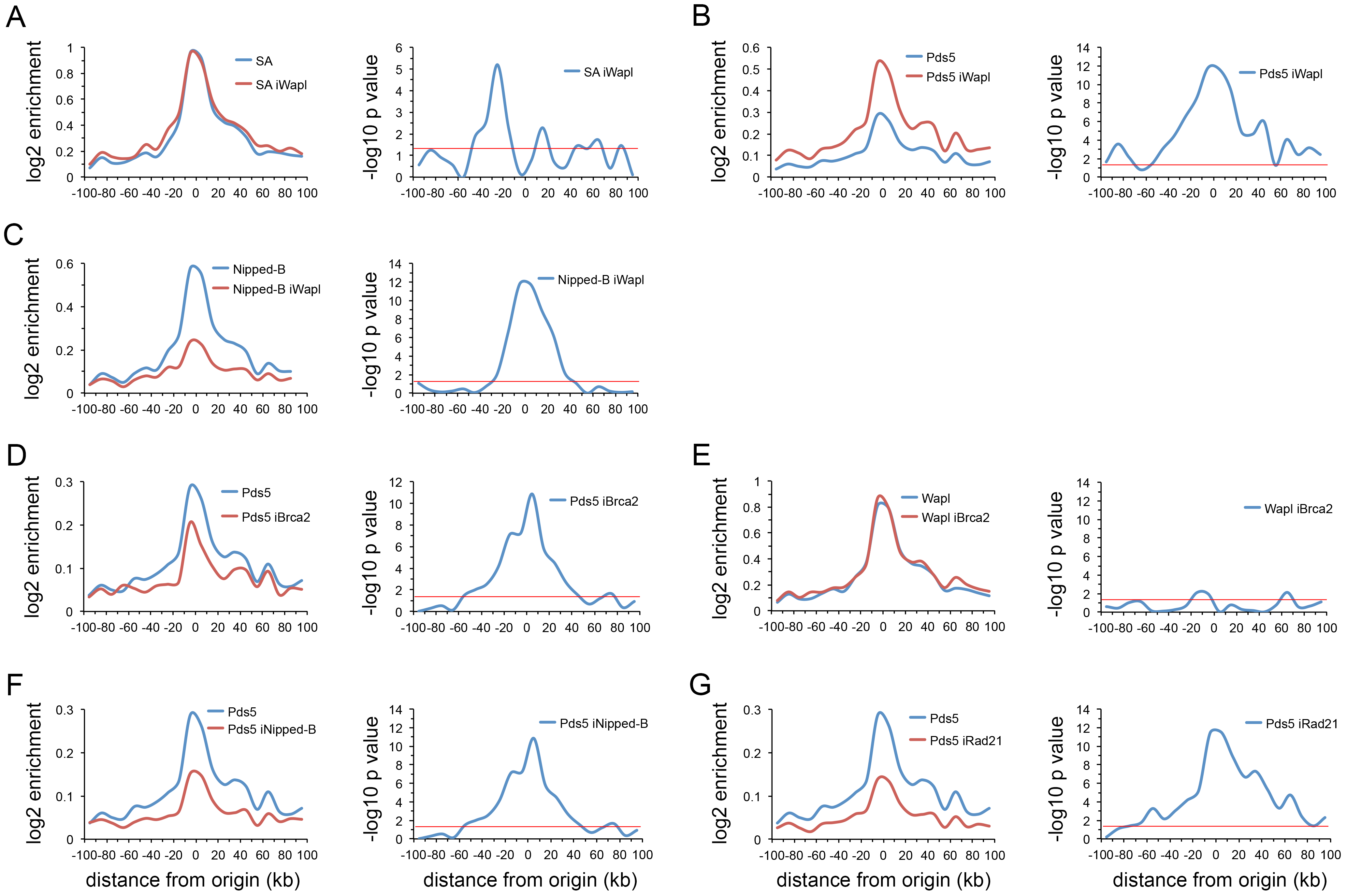

Supplement: S3 Fig — (A) Left panel is the SA distribution in mock-treated control cells (blue, SA) and cells depleted for Wapl (red, SA iWapl). Right panel is the -log10 p values of each bin for the difference in control versus the depletion calculated using the Wilcoxon signed rank test. (B) Same as A for the Pds5 distribution. (C) Same as A for the Nipped-B distribution. (D) Left panel is the Pds5 meta-origin distribution in control (blue, Pds5) cells and cells depleted for Brca2 (red, Pds5 iBrca2). Right panel shows the–log10 p values. (E) Same as D for Wapl distribution. (F) Left panel is the Pds5 distribution in control (blue, Pds5) cells, and cells depleted for Nipped-B (red, Pds5 iNipped-B). Right panel shows the -log10 p values. (G) Same as F except for Rad21 depleted cells. (TIF) [file pgen.1007225.s003.tif]

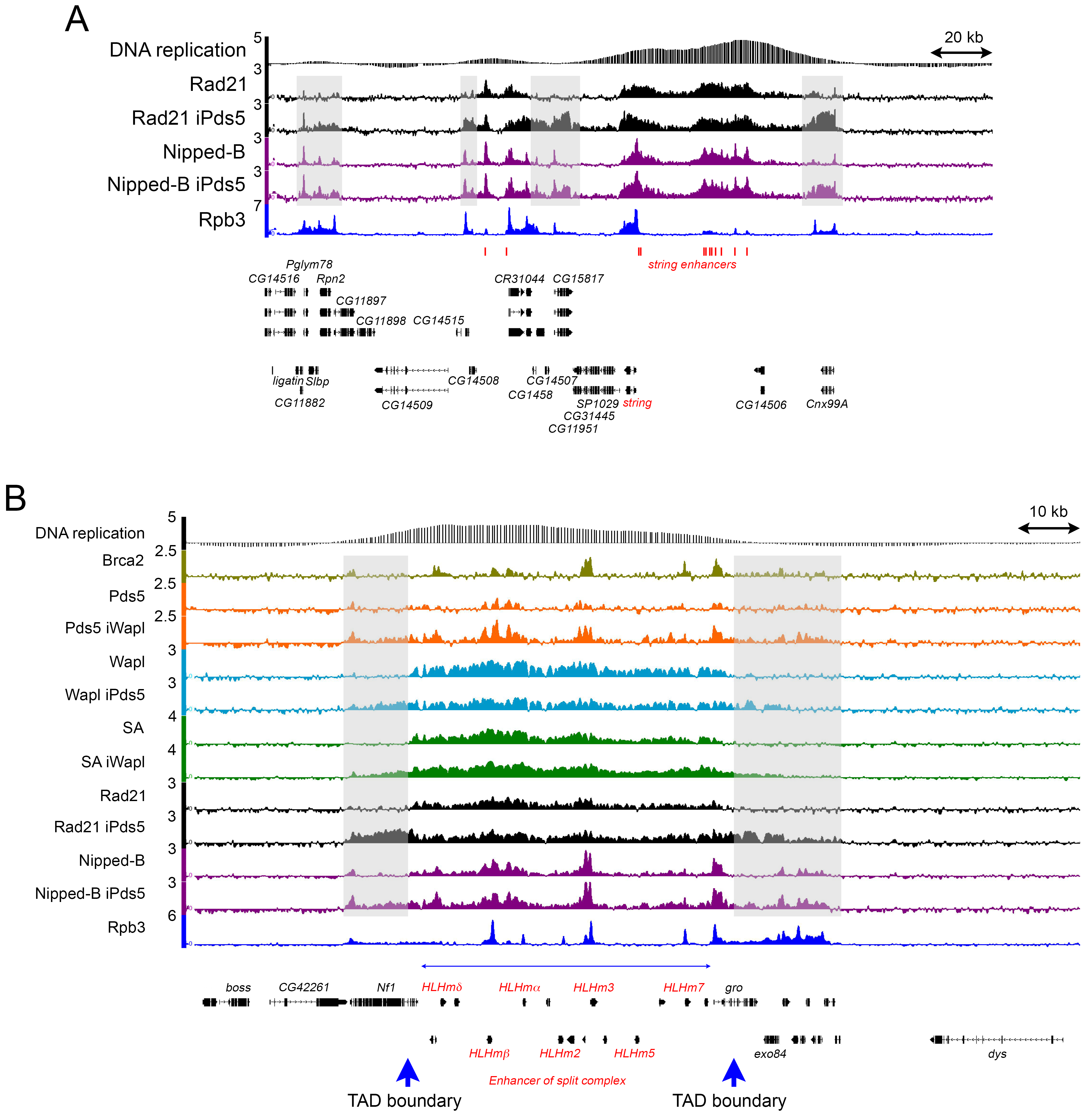

Supplement: S4 Fig — (A) Genome browser view of the string region showing skipping of spreading cohesin over inactive genes and intergenic regions. The string (Cdc25) gene and its active enhancers are labeled in red type. The top track is the early DNA replication pattern. The ChIP-seq tracks show the Rad21 (black) and Nipped-B (purple) binding patterns in control cells and cells depleted for Pds5 (iPds5). The bottom track shows the Rpb3 RNA polymerase pattern (blue). Shaded areas indicate regions of increased Rad21 and Nipped-B occupancy in Pds5-depleted cells. Pds5 depletion increases Rad21 and Nipped-B occupancy at the active gene cluster containing Pglym78 some 100 kb centromere-proximal (left) to the origin, but not with inactive genes such as CG14509 in the intervening region. Similarly, cohesin and Nipped-B increase substantially at the active Cxn99A gene to the right of the origin, but show only a modest increase in the intervening intergenic region. CG15817 is active but binds much less cohesin than some active genes located further from the origin. Pds5 depletion increases cohesin and Nipped-B at CG15817. (B) Enhancer of split gene complex showing expansion of cohesin domains beyond TAD (topologically associating domain) boundaries upon Pds5 or Wapl depletion. Enhancer of split genes are labeled in red, and the thin blue horizontal arrow just above the genes shows the extent of the gene complex. Wide vertical blue arrows below the genes indicate the TAD boundaries determined by 3C (chromosome conformation capture) [34]. The top track shows the early DNA replication pattern. The ChIP-seq tracks show the Pds5 (orange) pattern in control cells and cells depleted for Wapl (iWapl), the Wapl pattern (light blue) in control cells and cells depleted for Pds5 (iPds5), the SA (green) binding in control cells and cells depleted for Wapl (iWapl), the Rad21 (black) binding in control cells and cells depleted for Pds5 (iPds5), the Nipped-B (purple) pattern in control cells and cells deplete [file pgen.1007225.s004.tif]

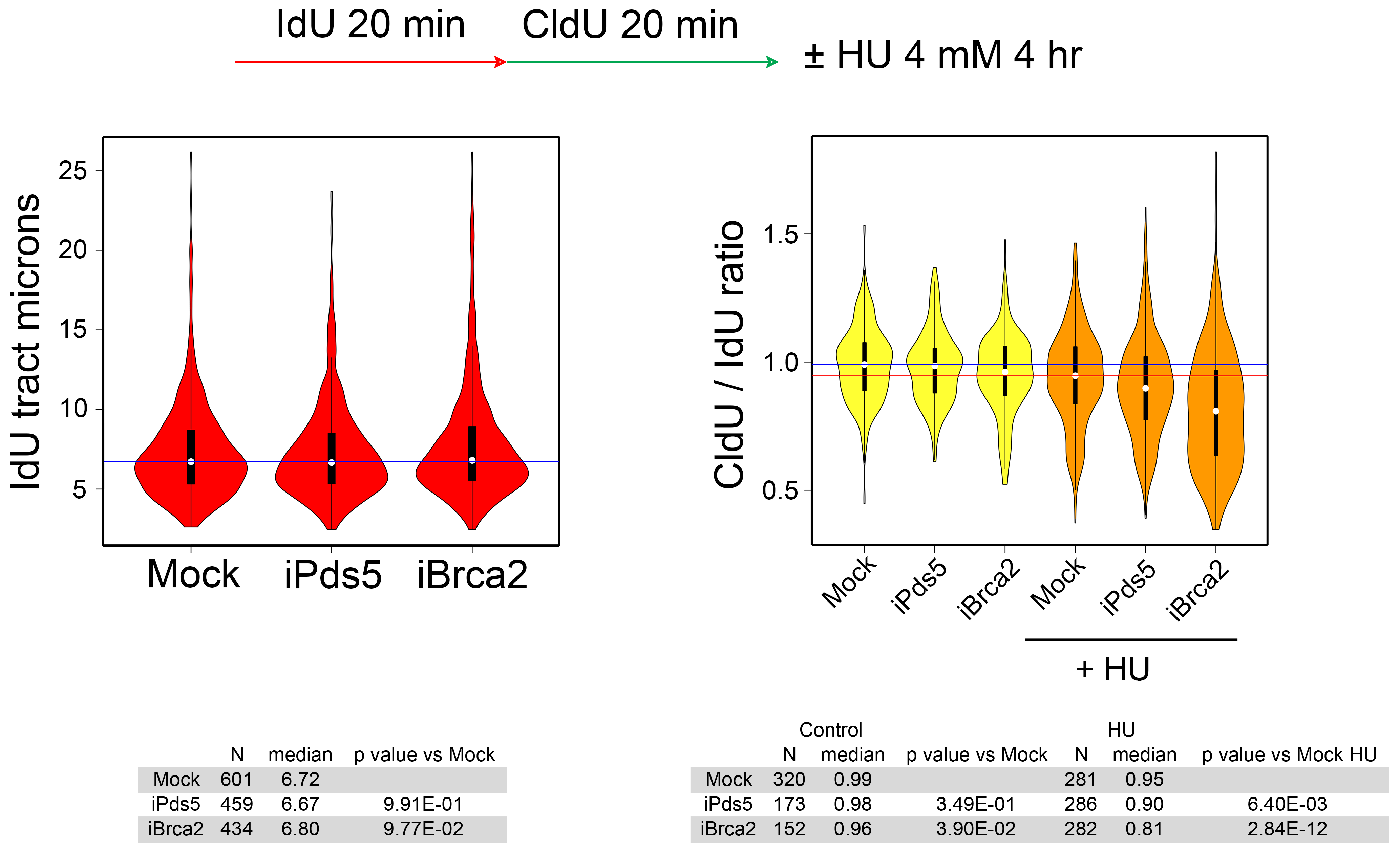

Supplement: S5 Fig — The top diagram shows the labeling scheme and the hydroxyurea (HU) treatment after CldU incorporation. The left panel shows the lengths of the IdU tracts in Mock control cells, and cells depleted for Pds5 (iPds5) or Brca2 (iBrca2). The blue line indicates the median tract length in Mock control cells. The IdU tracts were combined for the HU-treated and untreated cells, and only IdU tracts continuous with CldU tracts were measured. The table beneath the left panel gives the median tract lengths in microns for each group and the p values using the Wilcoxon test. The right panel shows the ratio of lengths of the CldU tracts to the connected IdU tracts. The blue line indicates the median ratio in Mock control cells not treated with HU, and the red line indicates the median ratio in Mock control cells treated with HU. The table beneath the right panel gives the median ratio for all groups and the p values for the indicated comparisons using the Wilcoxon test. Similar results were obtained in two other independent experiments. (TIF) [file pgen.1007225.s005.tif]

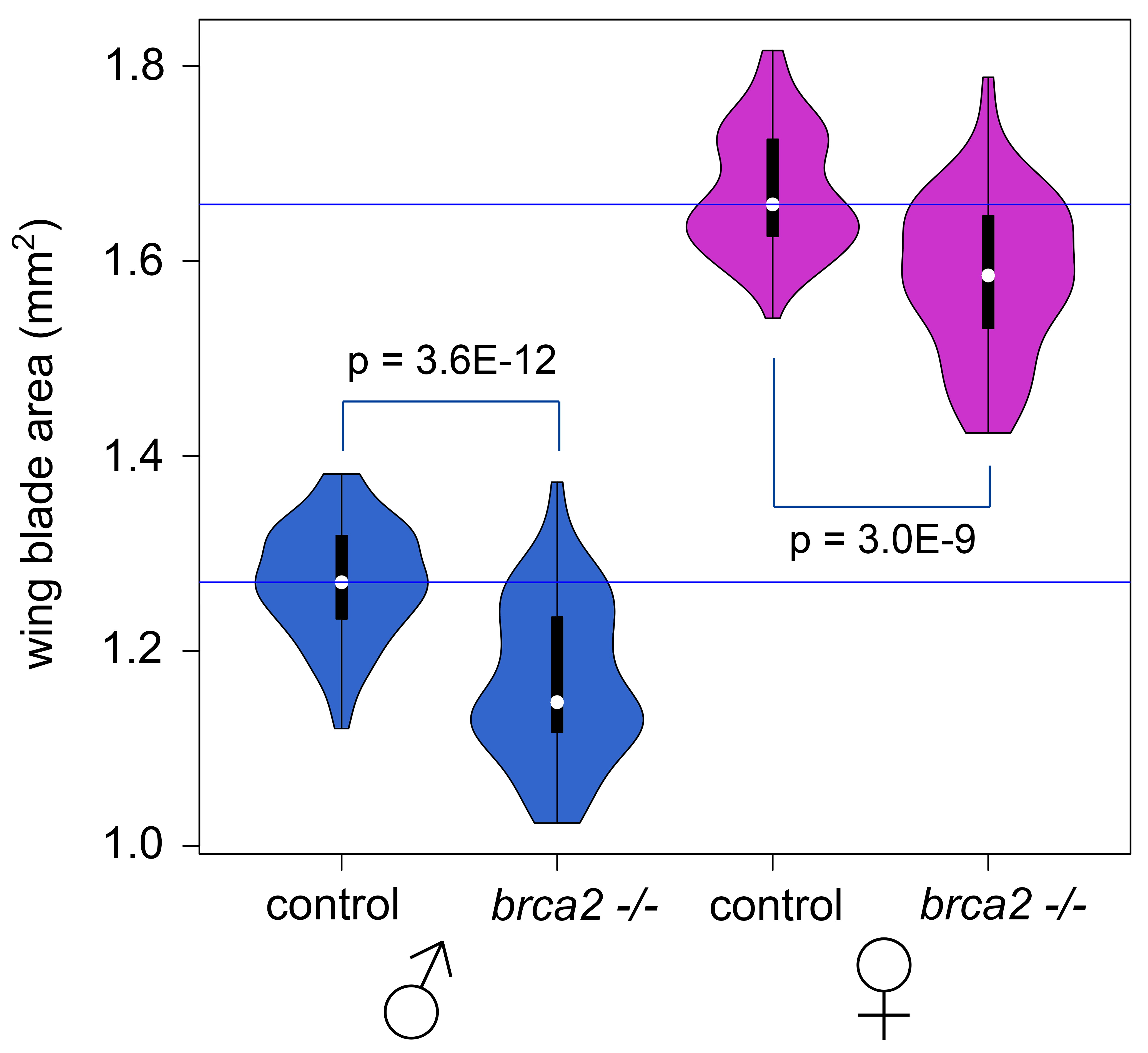

Supplement: S6 Fig — Violin plots show the distributions of adult wing blade areas from the indicated groups of flies grown at 25o. The brca2 -/- mutant wings are from ~20 homozygous brca2KO and ~40 brca2KO / brca256e flies and the control wings are from ~20 each from cn bw, Oregon R, brca2KO / cn bw and brca2KO / cn bw flies. The various mutant and control genotypes were combined to minimize the effects of genetic background differences that can influence growth. P values were calculated using the Wilcoxon test. Horizontal blue lines indicate the median wing areas for male and female control flies. (TIF) [file pgen.1007225.s006.tif]
